# Antiviral Activity and Underlying Mechanism of *Moslae herba* Aqueous Extract for Treating SARS-CoV-2

**DOI:** 10.3390/molecules30020387

**Published:** 2025-01-17

**Authors:** Yan Feng, Qiong Ge, Jian Gao, Zhuoying Wu, Yunyi Zhang, Haiyan Mao, Beibei Wu, Changping Xu

**Affiliations:** 1 Key Laboratory of Public Health Detection and Etiological Research of Zhejiang Province, Department of Microbiology, Zhejiang Provincial Center for Disease Control and Prevention, Hangzhou 310051, China; yfeng@cdc.zj.cn (Y.F.); qge@cdc.zj.cn (Q.G.); jgao@cdc.zj.cn (J.G.); hymao@cdc.zj.cn (H.M.); 2 Zhejiang Key Lab of Vaccine, Infectious Disease Prevention and Control, Zhejiang Provincial Center for Disease Control and Prevention, Hangzhou 310015, China; zhywu@cdc.zj.cn (Z.W.); yyzhang@cdc.zj.cn (Y.Z.); bbwu@cdc.zj.cn (B.W.)

**Keywords:** SARS-CoV-2, *Moslae herba*, HPLC-ESI-Q-TOF/MS, network pharmacology, molecular docking

## Abstract

Despite the widespread use of COVID-19 vaccines, there is still a global need to find effective therapeutics to deal with the variants of SARS-CoV-2. *Moslae herba* (MH) is a herbal medicine credited with antiviral effects. This study aims to investigate the antiviral effects and the underlying mechanism of aqueous extract of *Moslae herba* (AEMH) for treating SARS-CoV-2. The in vitro anti-SARS-CoV-2 activity of AEMH was evaluated using cell viability and viral load. Component analysis was performed by HPLC-ESI-Q-TOF/MS. The connection between COVID-19 and AEMH was constructed by integrating network pharmacology and transcriptome profiles to seek the core targets. The components with antiviral activities were analyzed by molecular docking and in vitro pharmacological verification. AEMH exerted anti-SARS-CoV-2 effects by inhibiting viral replication and reducing cell death caused by infection (IC_50_ is 170 μg/mL for omicron strain). A total of 27 components were identified from AEMH. Through matching 119 intersection targets of ‘disease and drug’ with 1082 differentially expressed genes of COVID-19 patients, nine genes were screened. Of the nine, the PNP and TPI1 were identified as core targets as AEMH treatment significantly regulated the mRNA expression level of the two genes on infected cells. Three components, caffeic acid, luteolin, and rosmarinic acid, displayed antiviral activities in verification. Molecular docking also demonstrated they could form stable bonds with the core targets. This study explored the antiviral activity and possible mechanism of AEMH for treating SARS-CoV-2, which could provide basic data and reference for the clinical application of MH.

## 1. Introduction

The coronavirus disease 2019 (COVID-19) pandemic, caused by severe acute respiratory syndrome coronavirus 2 (SARS-CoV-2), resulted in devastation to public health and the global economy. According to the World Health Organization, as of 17 November 2024, a total of 776,897,200 people were confirmed to be infected with SARS-CoV-2 worldwide, and above seven million patients died [1]. The swift development and application of vaccines made a great contribution to controlling the spread of COVID-19. However, along with the evolution of SARS-CoV-2, many variants appeared [2]. The variants may escape the humoral immune response and weaken the effectiveness of current vaccines, thereby resulting in new transmission. In terms of treatment, a variety of approaches, including the repurposing of previously adopted drugs, the development of novel virus-targeting antivirals, and the search for immune modulators and drug-capable host targets, have been used in the research of COVID-19 drugs [2,3]. Of these, Paxlovid, developed on the basis of the SARS-CoV-2 M^pro^ inhibitor nirmatrelvir (PF-07321332), had been approved and widely used for the treatment of COVID-19 [4]. Similarly to vaccines, antiviral drugs are also facing great challenges in dealing with new variants of SARS-CoV-2, especially the resistance mutants. For instance, most omicron variants were found to produce immune escape to therapeutic antibodies approved previously [5,6]. Hence, the constant search for efficient and safe compounds for the treatment of SARS-CoV-2 is still essential.

Chinese herbal medicine (CHM) is a unique resource in the medical system of China, and it has been used for the prevention and treatment of infectious diseases for thousands of years. After long-term practice, CHM was proven to be efficient and safe for the treatment of viral infections, such as influenza [7,8]. In terms of COVID-19, many researchers have provided evidence that CHM can not only help recovery of mild and ordinary cases but can also be an adjuvant treatment for severe cases [9,10,11]. Integration of CHM with standard care or Western medicine can significantly reduce mortality and improve the clinical symptoms and treatment outcomes of the patients [9,10]. As it is a whole consisting of multiple components, CHM exerts anti-SARS-CoV-2 effects through multiple mechanisms, including down-regulation of the expression of angiotensin-converting enzyme 2 (ACE2), blocking of the bond between ACE2 and the viral spike protein, and regulation of host immune function as well [12,13,14]. Hence, CHM, with the advantages of low toxicity and diverse activities, is an important source that could provide many potential compounds for further control of SARS-CoV-2 variants.

*Moslae herba* (MH) is the aboveground dry part of the *Mosla chinensis* Maxim or *M. chinensis.cv.* ‘Jiangxiangru’, a tomentose and aromatic plant belonging to the Lamiaceae family, is a traditional medicinal and edible herb mainly produced in southern China. Chinese pharmacopeia records that the herb has the effects of sweating and relieving the surface, as well as removing dampness for regulating the stomach. Clinically, it is widely used for treating summer-dampness cold, aversion to cold with fever, headache without sweating, abdominal pain, vomiting, and diarrhea. In modern Chinese medicine, MH is generally used as an ingredient of many prescriptions, such as Xinjia Xiangru Yin, Chaihu-Xiangru decoction, and Huanglian Xiangru decoction, which could be used for the treatment of common cold due to summer heat and dampness [15], acute upper respiratory infection in summer [16], as well as influenza [17]. Pharmacological research showed that MH has many biological activities, including antibacterial, antiviral, antioxidant, and anti-inflammatory. With the widespread application of chromatography and mass spectrometry technologies, the active components of MH were identified, which were roughly divided into volatile components and nonvolatile components [18]. So far, approximately 123 compounds have been identified from volatile components of MH. Most of them belong to flavonoids, terpenoids, phenolic acids, and phenylpropanoids [18]. Of these, flavonoids, a group of natural compounds derived from plants and with phenolic structures, were considered to have antiviral activities. The total flavonoids of MH inhibit the Influenza A virus by suppressing the NOX4/NF-κB/MLCK pathway [19]. Its main components, luteolin and apigenin, have antiviral effects on many viruses, including Japanese encephalitis virus (JEV), enterovirus 71 (EV71), respiratory syncytial virus (RSV), and herpes simplex virus 1 (HSV-1) [20,21,22,23]. For SARS-CoV-2, luteolin was considered an inhibitor of RNA-dependent RNA polymerase (RdRp) and the protease of 3CL [24,25], and a potent blocker of cell entry [26].

Previous research paid much attention to the volatile components of MH; however, the herb is traditionally processed as a decoction used for clinical. Whether the aqueous extract of *Moslae herba* (AEMH) has anti-SARS-CoV-2 activities, that is, whether MH could be a potential herb medicine used for the treatment of COVID-19, is the first question that we studied. Next, which components are present in AEMH and whether flavonoids exist and play an important role are still unknown. Therefore, component analysis of AEMH, as well as exploration of the active components, are essential to understanding the antiviral mechanism of AEMH. In this study, we first evaluated the anti-SARS-CoV-2 activities of AEMH and found that AEMH suppressed viral replication intracellularly. Then, the material basis of AEMH was analyzed, and a total of 26 components were identified. Integrating network pharmacology and transcriptional profiling, two core regulators were screened and identified. Finally, the active components were screened and verified by molecular docking and in vitro pharmacological experiments. Our results could provide not only a reference for the clinical applications of MH but also a valuable insight for developing antiviral drugs with herbal medicines.

## 2. Results

### 2.1. In Vitro Antiviral Effects of AEMH on SARS-CoV-2

**Figure 1:**
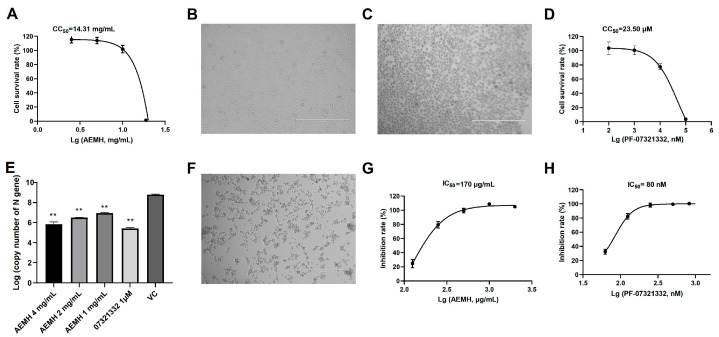
Evaluation of in vitro anti-SARS-CoV-2 activities of AEMH. (**A**) Cytotoxicity induced by AEMH on Vero-E6 cells. (**B**) Normal Vero-E6 cell morphology. (**C**) Vero-E6 cell morphology treated by 20 mg/mL AEMH. (**D**) Cytotoxicity induced by Nirmatrelvir on Vero-E6 cells. (**E**) SARS-CoV-2 viral loads on cells with or without AEMH treatment. Vero-E6 cells were infected with 100 TCID_50_ of SARS-CoV-2 and treated with different concentrations of AEMH or Nirmatrelvir (1 μM) for 48 h. Viral loads were tested by quantitative real-time PCR (qRT-PCR) targeting N gene of SARS-CoV-2. **, significant with *p* < 0.01 compared to VC. (**F**) Omicron-induced CPE on Vero-E6 cells. (**G**) AEMH inhibited cell deaths induced by SARS-CoV-2 infection. (**H**) Nirmatrelvir inhibited cell deaths induced by SARS-CoV-2 infection.

To avoid cytotoxicity in antiviral experiments, the maximum non-toxic concentration (MNTC) of AEMH was first determined on Vero-E6 cells. When 10 mg/mL of AEMH was inoculated, the cell survival rates were 101.94 ± 4.83% (Figure 1A), and the cells did not differ in morphology from normal E6 cells (Figure 1B). A large number of cells died when the concentration of AEMH increased to 20 mg/mL (Figure 1C), and the cell survival rate sharply dropped to 1.44 ± 1.27% (Figure 1A). Hence, the MNTC of AEMH was identified as 10 mg/mL, and the CC_50_ was calculated as 14.31 mg/mL. Also, the MNTC and CC_50_ of positive control Nirmatrelvir (PF-07321332) were tested as 1 μM and 23.50 μM (Figure 1D).

To evaluate the in vitro anti-SARS-CoV-2 activities of AEMH, we compared the viral loads of infected cells with or without AEMH treatment and also investigated the inhibition of AEMH on cell death caused by infection. When the cells were treated with three concentrations of AEMH for 48 h after infection, the viral loads of the treatment groups were all markedly dropped compared to the VC group (*p* < 0.01), with 67~782 fold reductions (Figure 1E). CPE occurred on about 80% of cells at 72 h post-infection (Figure 1F), but it was efficiently inhibited by AEMH treatment. Cell survival rates demonstrated that AEMH treatment significantly inhibited cell death caused by viral infection (Figure 1G), and the inhibition rates have a dose–effect relationship with the concentration of AEMH. The median inhibitory concentration (IC_50_) of AEMH and Nirmatrelvirw was calculated as 170 µg/mL and 80 nM, respectively (Figure 1G,H).

### 2.2. Components Identified from AEMH

**Figure 2:**
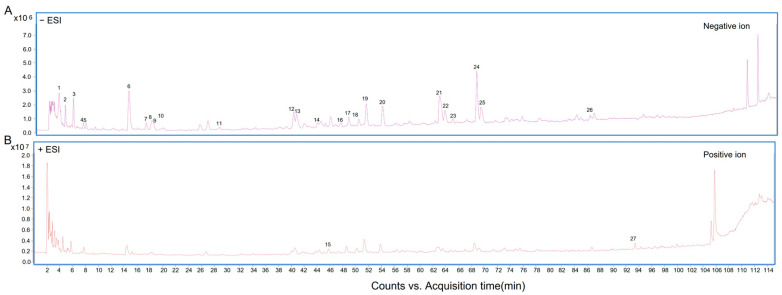
Components analysis performed by HPLC-ESI-Q-TOF/MS. (**A**) Negative ion chromatogram of AEMH. (**B**) Positive ion chromatogram of AEMH.

For investigating the antiviral mechanism, the material basis of AEMH was analyzed by HPLC-ESI-Q-TOF/MS. The positive ion and negative ion chromatograms are displayed in Figure 2. By comparing with the database, a total of 27 components were identified in AEMH (Table 1). As one out of the 27, that is, the compound with molecular formula C18H28O9, can not be exactly distinguished, only 26 components were used for the research of network pharmacology.

### 2.3. Screening of Targets Related to COVID-19 and AEMH

**Figure 3:**
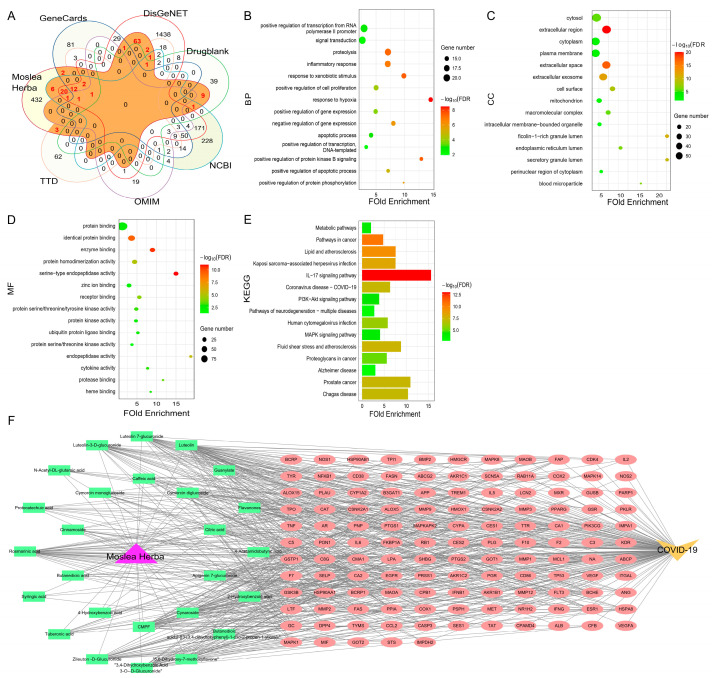
Screening and analysis of targets related to COVID-19 and AEMH. (**A**) Venn plot constructed based on COVID-19-related targets retrieved from six databases and the related targets of 26 components identified from AEMH. (**B**–**D**) GO enrichment analysis of 126 intersection genes. The top 15 items involved in BP (**B**), CC (**C**), and MF (**D**). (**E**) The top 15 pathways enriched in KEGG analysis (FDR < 0.05). (**F**) AEMH-components-targets-COVID-19 network diagram.

COVID-19-related targets were searched through six databases. There are 61, 214, 40, 540, 1843, and 109 targets obtained from the DrugBank, GeneCards, OMIM, NCBI, DisGeNET, and TTD databases, respectively. For screening the AEMH-related targets, 26 components identified by HPLC-ESI-Q-TOF/MS were imported to three databases (PharmMapper, SEA, and TCMSP), and 558 related targets were retrieved. Then, by matching COVID-19-related targets with components-related targets, a total of 126 targets were found in the intersection, namely, the common targets of ‘disease and drug’ (Figure 3A).

Further, we performed Gene Ontology (GO) and Kyoto Encyclopedia of Genes and Genomes (KEGG) analysis based on the 126 intersections targets. From the DAVID database, GO enrichment analysis screened 177 GO items (FDR < 0.05). Of these, 119 items were involved in a Biological Process (BP), while only 20 were involved in a Cellular Component (CC), and 38 were involved in a Molecular Function (MF). The bubble charts showed the top 15 pathways that the 126 genes were enriched in BP, CC, and MF (Figure 3B–D). KEGG analysis showed that the 126 genes were involved in 124 pathways (FDR < 0.05). The top 15 enriched pathways are shown in Figure 3E. Of the 124 pathways, the IL-17 signaling pathway is most significantly involved. In addition, the ‘drug-components-targets-disease’ network was constructed using one medicine (AEMH), 26 components, 126 targets, and the disease COVID-19. The network was composed of 154 nodes and 629 edges (Figure 3F).

### 2.4. Screening and Verification of Core Targets

**Figure 4:**
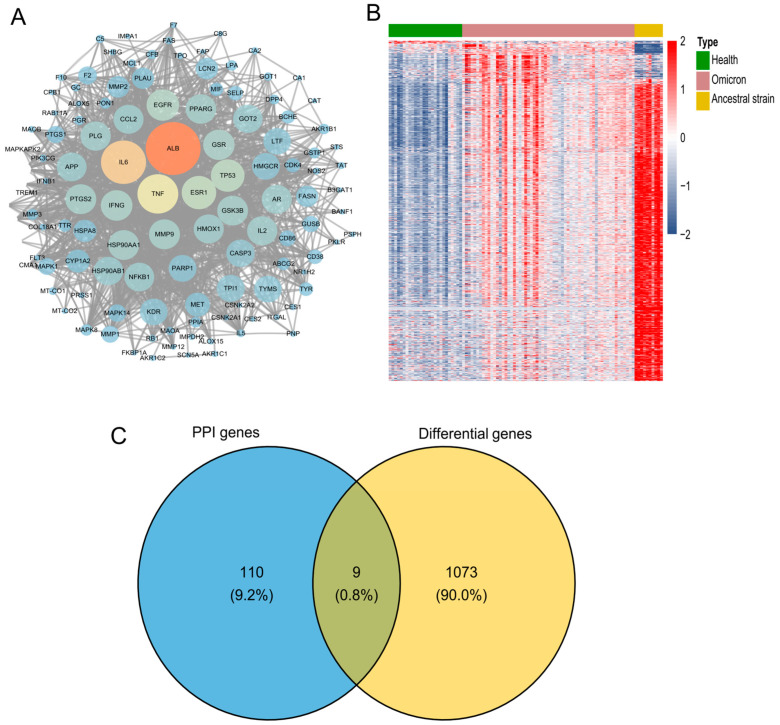
Screening of core targets. (**A**) PPI network diagram. (**B**) Heat map plotted with differentially expressed genes of patients in comparison with healthy donors. (**C**) Venn plot constructed based on 119 genes obtained in PPI with 1082 differential expression genes of patients infected with omicron.

For seeking the core targets, the 126 intersection genes were input into the STRING database to construct a Protein–Protein Interaction (PPI) network (Figure 4A). The PPI network contained 119 nodes (119 out of 126 genes) and 1341 edges. Figure 4B showed the global change in the transcriptome between patients infected with Omicron or ancestral strains and health donors, and 1082 genes were found to be differentially expressed in omicron-infected patients [27]. Then, 119 genes obtained from PPI were matched with the 1082 differential expression genes. Nine genes, as shown in the Venn plot, including Heme oxygenase 1 (HMOX1), Lipocalin 2 (LCN2), Monoamine oxidase B (MAOB), Myeloid Cell Leukemia 1 (MCL1), Matrix metallopeptidase 1 (MMP1), Purine nucleoside phosphorylase (PNP), selectin P (SELP), Triosephosphate isomerase 1 (TPI1), and MAPK-activated protein kinase 2 (MAPKAPK2), were found in the intersection region (Figure 4C).

**Figure 5:**
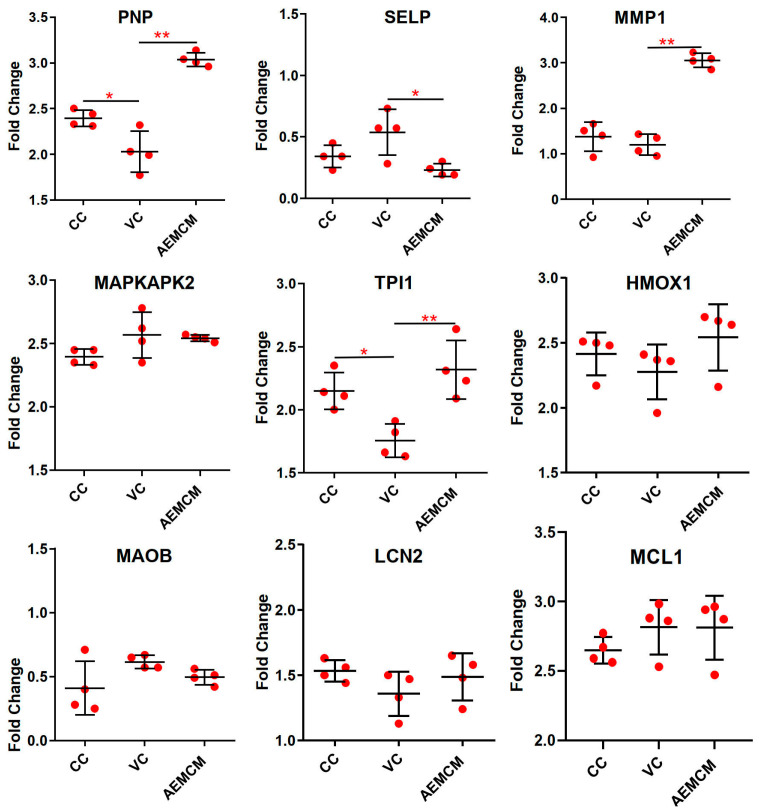
Relative mRNA expression level of the selected nine genes. Each gene contains data from the CC-, VC-, and AEMH-treated groups. Experiments were performed in four replicates. *, significant with *p* < 0.05, **, significant with *p* < 0.01.

The nine genes were validated by qRT-PCR and the mRNA level was presented with fold-change based on Ct value. Of the nine, the mRNA level of two genes, PNP and TPI1, was markedly downregulated in the VC group, and then significantly more transcribed in the AEMH-treated group (Figure 5). A significant difference was also observed in the relative mRNA expression level of the MMP1 gene between the VC- and the AEMH-treated groups, but there was no difference between the VC and the CC groups. Hence, the PNP and TPI1 were selected as the key regulators.

### 2.5. Screening and Identification of Active Components

**Figure 6:**
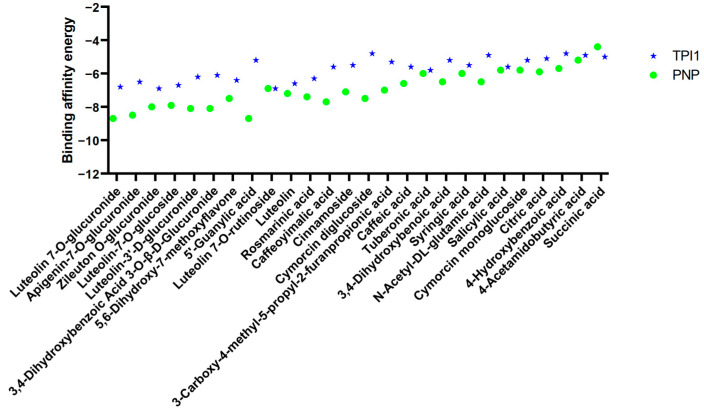
Binding affinity energies between core targets and 26 components. The binding affinity energies were evaluated by the docking scores of components and core targets.

For seeking the single components with anti-SARS-CoV-2 activity, the connection between 26 components and proteins of two core targets was analyzed by molecular docking. The binding affinity energies, as shown in Figure 6, demonstrated a total of 20 components with binding energies lower than −6 Kcal/mol with PNP, and the lowest one was Luteolin-7-O-glucuronide (−8.7 Kcal/mol). The overall binding energies between components and TPI1 were higher than those with PNP, and the lowest one was Luteolin-7-O-rutinoside, with a binding energy of −6.9 Kcal/mol.

**Figure 7:**
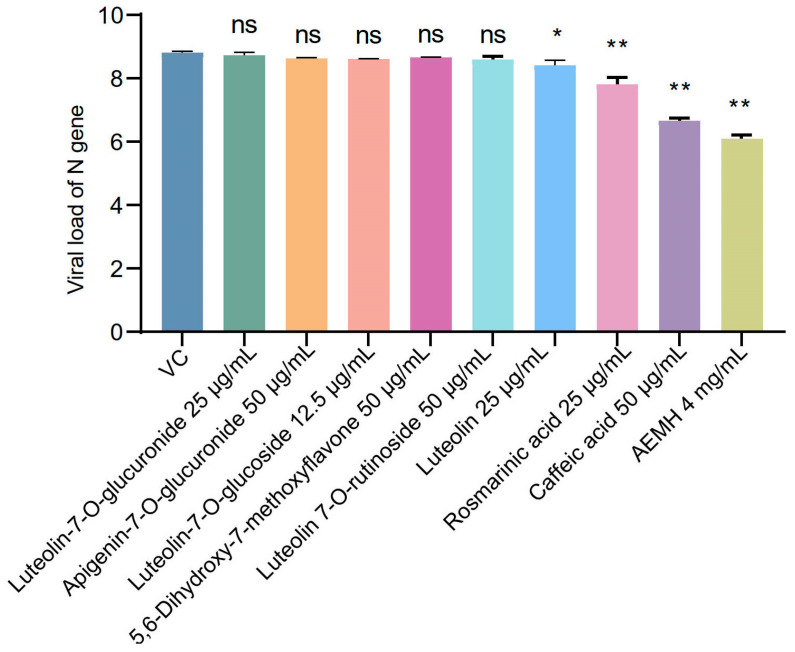
In vitro pharmacological verification of eight selected components. Vero-E6 cells were infected with 100 TCID_50_ of SARS-CoV-2 and then treated with each component at MNTC or 4 mg/mL of AEMH for 48 h. Viral loads were tested by qRT-PCR targeting N gene of SARS-CoV-2. *, significant with *p* < 0.05 compared to VC. **, significant with *p* < 0.01 compared to VC, ns, non-significant.

**Figure 8:**
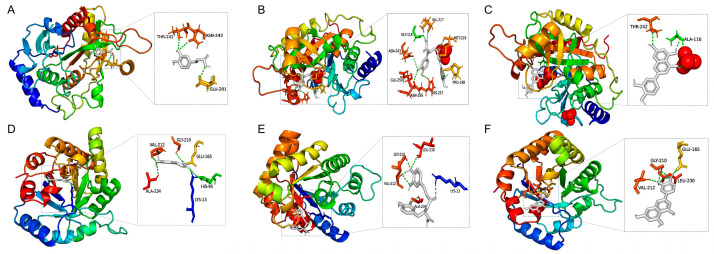
Binding patterns between three verified components and two core targets. Binding pattern between PNP to caffeic acid (**A**), rosmarinic acid (**B**), and luteolin (**C**). Binding pattern between TPI1 to caffeic acid (**D**), rosmarinic acid (**E**), and luteolin (**F**).

Since not all the components are available, only eight with binding energies lower than −6 Kcal/mol were selected to perform in vitro pharmacological verification (Figure 7). The MNTC was tested as 25 µg/mL for Luteolin 7-O-glucuronide, Luteolin, and Rosmarinic acid, 50 µg/mL for Apigenin-7-O-glucuronide, 5,6-Dihydroxy-7-methoxyflavone, Luteolin 7-O-rutinoside, and Caffeic acid, and only 12.5 µg/mL for Luteolin-7-O-glucoside. After 48 h post-infection, viral loads of the luteolin-, rosmarinic acid-, and caffeic acid-treated groups remarkably dropped compared to the VC, indicating the three components have in vitro antiviral effects on SARS-CoV-2 at MNTC. Additionally, molecular docking between three verified components with the proteins of PNP or TPI1 was conducted, and the results are shown in Figure 8.

## 3. Discussion

In this study, AEMH displayed significant in vitro antiviral effects against SARS-CoV-2 by inhibiting viral replication intracellularly and cell death caused by viral infection. MH or compounds containing MH have been employed to treat fever, influenza, and pneumonia in China for a long time [15,16,17]. Previous studies demonstrated that MH and its active components, for example, total flavonoids, can inhibit influenza A virus-induced CPE in MDCK cells and also reduce viral loads in the lungs of infected mice [28,29], which is similar to the results of our study.

As herbal medicine is a multi-component substance, it is necessary to clarify its material basis in order to study the mechanism of antiviral action. The active components of MH have been extensively studied [18,30]. Duan et al. summarized 123 components identified from *Mosla chinensis* Maxim and *M. chinensis.cv.* ‘Jiangxiangru’, and most of them are flavonoids, terpenoids, phenolic acids, and phenylpropanoids [18]. Also, Wang et al. identified 69 chemical constituents of *M. chinensis.cv.* ‘Jiangxiangru’ by UPLC-LTQ-Orbitrap-MS [30]. Compared with previous studies that prepared samples with organic solvents, such as ethanol and methyl alcohol, we analyzed the material basis of MH aqueous extract since traditional CHM is usually used as a decoction. In total, 27 components were identified in our study. Of these, nine components, including citric acid, succinic acid, caffeic acid, 3,4-Dihydroxybenzoic acid, 4-Hydroxybenzoic acid, Syringic acid, rosmarinic acid, luteolin, and 5,6-Dihydroxy-7-methoxyflavone, were reported, while another 17 were identified for the first time, as far as we know. The flavonoids Luteolin and Apigenin were considered representative of MH [28], but we identified only luteolin from AEMH. In addition, eight organic acids were identified; of these, citric acid, succinic acid, caffeic acid, and rosmarinic acid were also determined from *M. chinensis.cv.* ‘Jiangxiangru’ [30].

Due to the complex composition, the action of CHM is considered to be multi-target and multi-pathway. Therefore, it is a challenge to explore the possible mode of action of CHM. In recent years, the development of bioinformatics has provided a novel and effective strategy for the investigation of the antiviral mechanism of CHM. During the epidemic of COVID-19, many researchers investigated the potential mechanism and active components of herbal medicine for treating SARS-CoV-2 through network pharmacology combined with molecular docking, such as Andrographis paniculata and Fuzheng Yugan Mixture [31,32]. In this study, we began by retrieving targets related to COVID-19 and AEMH and then obtained the common targets of ‘drug and disease’. Secondly, the ‘drug–disease’ targets were re-intersected with differentially expressed genes of COVID-19 patients [27]. As the transcriptome data were generated from real patients and health donors, the re-intersection could narrow the range of core targets and further increase the accuracy of target screening. As a result, nine genes were found based on two screenings. Subsequently, PNP and TPI1 were identified as core regulators by comparing the relative mRNA expression levels amongst the VC-, CC-, and AEMH-treated groups.

PNP is a key enzyme involved in purine degradation and salvaging, which plays an important role in regulating levels of purine metabolites. It mainly functions to phosphorylate inosine and deoxyinosine to hypoxanthine and to phosphorylate guanosine and deoxyguanosine to guanin, which is finally converted to uric acid [33]. In terms of pathogen infection, recent research reported that PNP-1 negatively regulates the expression of genes that are induced by bacterial infection [34], and PNP from mycobacterium tuberculosis is an attractive target for tuberculosis treatments [35]. The relationship between PNP and viral infection has been less studied. However, patients with PNP deficiency usually exhibit a high incidence of viral infection since impaired PNP results in marked T lineage lymphopenia and damage to the immune system. For instance, Bandar et al. reported a PNP deficiency patient with recurrent infections with EBV and varicella [36]. As virus replication is completely reliant on host nucleotides, whether PNP plays an indirect antiviral role by regulating purine metabolism and host immunity remains to be further studied. Triosephosphate isomerase 1 (TPI1), a key enzyme in the glycolytic pathway, catalyzes the rapid conversion between dihydroxyacetone phosphate and 3-phosphoglyceraldehyde. The relationship between the expression of TPI1 and virus infection or anti-infectious immunity is unclear. The only report we retrieved was that TPI was considered to be significantly upregulated during RSV infection [37]. Although PNP and TPI1 were screened as the core regulators of AEMH against SARS-CoV-2 through molecular docking, the detailed molecular mechanism should be further investigated.

In order to explore the single component with anti-SARS-CoV-2 activity in AEMH, the 26 components were molecularly docked with PNP and TPI1, respectively. The binding energies indicated that 20 components could form stable bonds with the protein of PNP or TPI1, thereby exerting antiviral activity. Of the 20 components, only eight were selected to do in vitro pharmacological verification. Three components, including two organic acids (caffeic acid and rosmarinic acid) and a flavonoid (luteolin), displayed antiviral effects at their MNTC.

Caffeic acid, a phenolic compound found in most plants, was reported to have significantly inhibitory effects against HCoV-NL63, Influenza virus, Hepatitis C virus, HSV, and ILHV [38,39,40,41,42]. Previous studies have put forward the idea that polyphenol may have the potential to combat COVID-19 and have analyzed the binding ability between caffeic acid and its derivatives with SARS-CoV-2 by molecular docking [43]. Of the three components verified in our study, caffeic acid displayed the highest anti-SARS-CoV-2 activity at the MNTC, which proved the above point of view. Rosmarinic acid, an ester of caffeic acid and 3,4-dihydroxyphenyllactic acid, has a wide range of pharmacological properties, such as antiviral, anti-inflammatory, and antioxidant properties. Evidence from in vitro or in vivo studies demonstrated that rosmarinic acid possesses antiviral activities on the Chikungunya virus, Monkeypox, influenza virus, and EV71 viruses [44,45,46,47]. For SARS-CoV-2, crystal structure elucidated that rosmarinic acid can form a complex with SARS-CoV-2 M^pro^ [48]. In an in vitro antiviral experiment, rosmarinic acid exerted anti-SARS-CoV-2 activities with an IC_50_ of 25.47 ng [49]. Rosmarinic acid significantly inhibited the replication of SARS-CoV-2 in our study, which is similar to the previous reports. Luteolin, a nontoxic and non-mutagenic dietary flavonoid existing in a variety of fruits and vegetables, was considered a promising antiviral agent for many viruses. For instance, Luteolin inhibited RSV replication by regulating the miR-155/SOCS1/STAT1 signaling pathway [22], inhibited HSV-1 through enhancing type I interferon production [23], and conferred a survival protection of 91.67% from the lethal EV71 challenge in newborn mice [21]. For SARS-CoV-2, luteolin was reported to display antiviral activities through binding with RdRp, 3CL^pro^, and spike proteins [24,25,26]. Most of the publications were performed by molecular docking or enzymatic inhibition assay, but few pieces of research have proved the antiviral effects of luteolin using a live virus strain. Our results filled the gap by confirming the anti-SARS-CoV-2 activity of luteolin with a real omicron strain. However, Apigenin-7-O-glucoside and Luteolin-7-O-glucoside, which exhibited lower binding energies with PNP in our study and have been reported to show activity against RdRp [50], did not demonstrate any antiviral activity at the MNTC against live SARS-CoV-2.

The limitation of this study is that the in vivo anti-SARS-CoV-2 activities of AEMH were not assessed because of the absence of the Animal Biosafety Level 3 Laboratory (ABSL-3), and, also, the mechanisms of caffeic acid, rosmarinic acid, and luteolin need to be further studied.

## 4. Materials and Methods

### 4.1. Medicines

*Moslae herba* used in this study is the aboveground part of *Mosla chinensis* Maxim. The herb was collected and manufactured by Zhejiang Chinese Medical University Medical Pieces., Ltd., Hang Zhou, China. A crude slice of the herb was provided and authenticated by Prof. Qiaofeng Wu at Zhejiang Chinese Medical University (ZCMU). The AEMH was prepared by the Pharmaceutical Preparation Department of ZCMU. Briefly, the herb was boiled and refluxed in 30 volumes of water (~3.33% *w*/*v*) for 1 h. The extraction process was repeated twice followed by filtration. Then, the filtered liquid was concentrated under reduced pressure to 50 mL and was determined to contain 1 g/mL of crude herb. The pieces of the herb were stored at the Basic Chemistry Laboratory of ZCMU, and the number was 150801. The Nirmatrelvir powder (PF-07321332) (MedChemExpress, Monmouth Junction, NJ, USA) was dissolved in dimethyl sulfoxide (DMSO), making the initial concentration 1 mM. CP-100356 hydrochloride (MedChemExpress, Monmouth Junction, NJ, USA), dissolved to 1 mM with DMSO, was used as an efflux inhibitor.

Rosmarinic acid (CAS:20283-92-5) standards were purchased from BBI Life Science Corporation, China. The caffeic acid (CAS: 331-39-5) standard was purchased from the National Institutes for Food and Drug Control. The luteolin 7-O-glucuronide (CAS: 29741-10-4) standard was purchased from Baoji Herbest Bio-Tech Co., Ltd., Baoji, China. The 5,6-Dihydroxy-7-methoxyflavone (CAS: 29550-13-8), Apigenin-7-O-glucuronide (CAS: 29741-09-1), Luteolin-7-O-glucoside (CAS: 5373-11-5), and Luteolin 7-O-rutinoside (CAS: 20633-84-5) standards were purchased from Chengdu Desite Biotech Co., Ltd., Chengdu, China. The luteolin (CAS: 491-70-3) standard was purchased from Shanghai Macklin Biochemical Co., Ltd., Shanghai, China. All the standards were dissolved with DMSO, making the initial concentration 10 mg/mL, and stored at −20 °C.

### 4.2. Viruses and Cells

The SARS-CoV-2 strain SARS-CoV-2/VeroE6/DSh/2021ZJ25 (Omicron/B.1.1/EPI_ISL_ 12040149) was isolated from a throat swab of a suspected COVID-19 patient and kept at −80 °C in Zhejiang CDC [51]. All experiments involving live viruses were performed in a Biosafety Level 3 (BSL-3) laboratory. The initial titer of the virus stock was 10^5.6^ TCID_50_/0.1 mL. Vero-E6 cells were cultured in Minimum Essential Medium supplemented with 10% fetal bovine serum, 1% L-glutathione, and 1% penicillin–streptomycin solution.

### 4.3. Cytotoxicity of Medicines

The cytotoxicity of AEMH and Nirmatrelvir, as well as the eight components that were used for in vitro pharmacological verification, were determined on Vero-E6 cells by the cell counting Kit-8 (CCK-8) (Beyotime Biotechnology, Shanghai). After the cell monolayer formed in a 96-well plate, the growth medium was decanted and displaced by 100 μL of different concentrations of medicine. Each concentration was performed in five replicates. A total of 100 μL of maintenance medium (MM), instead of medicine, was added to the cell control. After incubation for 72 hrs, the medicine solution was removed and 100 μL of MM containing 10 μL of CCK-8 was added to the cells. The plates were re-incubated for 1.5 h at 37 °C and the optical density value was measured at 450 nm after incubation. The cell survival rate and the CC_50_ were calculated by the following formulas. The highest concentration with a cell viability rate higher than 90% was determined as the MNTC of the medicine.

Cell survival rate (%) = (OD of drug group − mean OD of blank)/(mean OD of CC −
mean OD of blank) × 100%.
Distance rate = (50% − cell survival rate < 50%)/(cell survival rate > 50% − cell survival
rate < 50%)
CC50 = 10∧{log (drug concentration with survival rate < 50%) − distance rate × log
(dilution ratio)}

### 4.4. In Vitro Antiviral Verification Experiment

The in vitro antiviral activity was validated using both a gene amplification reduction assay and a cell viability rate assay. In the gene amplification reduction experiments, Vero-E6 seeded in 24-well plates were infected with 100 TCID_50_ of SARS-CoV-2 strain for 1 h. The cells were then washed twice with PBS to remove the unbound viral particles. Then, the cells were treated with 1 mL of AEMH (4, 2, and 1 mg/mL), Nirmatrelvir (1 μM), or seven chemical compounds with MNTC, respectively. Each concentration of AEMH and Nirmatrelvir was performed in five replicates, while the chemical compounds were performed in triplicates. For the virus control (VC) group, the infected cells were treated with 1 mL of MM. After 48 h of incubation at 37 °C in a 5% CO_2_ incubator, viral RNA was extracted from each group, and viral load was tested by qRT-PCR. The antiviral effects were confirmed by comparing the viral loads between the VC- and medicine-treated groups.

In regard to the cell viability rate assay, vero-E6 cells were infected with 100 TCID_50_ SARS-CoV-2 in 96-well plates for 1 h at 37 °C. After infection, the virus fluid was decanted and 100 μL of different concentrations of medicine was added to the cells. For VC, the cells were covered with 100 µL MM without medicine after infection. Each concentration was performed in five replicates. After incubation for 72 h, the cell viability rate was measured by the same assay as for the cytotoxicity testing. The IC_50_ of medicine was calculated using Graphpad software (version 6.01). The results are shown as the mean ± standard deviation.

### 4.5. HPLC-ESI-Q-TOF/MS Conditions

Component analysis of AEMH was performed on an Agilent1260-6530 QTOF system (Agilent, CA, USA). Separation was carried out on an Agilent ZORBAX Eclipse XDB-C18 column (5 µm, 4.6 ∗ 250 mm) (Agilent, Santa Clara, CA, USA) with an injection volume of 10 μL. (The aliquot of 1 mL AEMH (1 g/mL) was diluted with 25 mL water and then processed with a 0.22 µm filter). Acetonitrile was used as mobile phase A and 0.1% formic acid solution as mobile phase B. The flow rate was 1 mL/min. The column temperature was 30 °C and the detection wavelengths were 254 nm and 360 nm (full wavelength enabled). The mass spectrometer was operated in the positive and negative ion modes. Other parameters were set as follows: capillary voltage (Vcap), 3.5 KV; nebulizer pressure, 35 psig; capillary outlet voltage (Fragmentor), 65 V; skimmer voltage, 65 V; dring Gas flow rate, 8 L/min; collision energy, 20 V; scanning range, 100~1700 *m*/*z*. MS data were acquired by Aglient Technologies MassHunter Acquisition (Version B.05.00) and processed using Qualtitave Analysis (Version 10.0).

### 4.6. Network Pharmacology

#### 4.6.1. Screening of the SARS-CoV-2-Related Targets and the AEMH-Related Targets

SARS-CoV-2-related targets were screened from six databases, including DrugBank (https://www.drugbank.ca, accessed on 4 September 2024), GeneCards (https://www.genecards.org/, accessed on 4 September 2024), OMIM (https://www.omim.org/, accessed on 4 September 2024), NCBI (https://www.ncbi.nlm.nih.gov/, accessed on 4 September 2024), DisGeNET (https://www.disgenet.com/, accessed on 4 September 2024), and TTD (http://db.idrblab.net/ttd/, accessed on 4 September 2024) with the keywords ‘COVID-19’, ‘Coronavirus 2019’, ‘Novel coronavirus’, or ‘Coronavirus disease 2019’. Of the targets searched from GeneCards, those with scores ≥10.0 were selected as SARS-CoV-2-related targets.

A total of 26 components identified by the HPLC-ESI-Q-TOF/MS were used for the screening of AEMH-related targets. Briefly, the components that were downloaded from PUbChem (https://pubchem.ncbi.nlm.nih.gov/, accessed on 5 September 2024) in SDF format were imported into the PharmMapper server database (http://www.lilab-ecust.cn/pharmmapper/, accessed on 5 September 2024) for target searching. Genes with Norm Fit ≥ 0.9 were selected as targets. Another two databases, SEA (https://sea.bkslab.org/, accessed on 5 September 2024) and TCMSP (https://old.tcmsp-e.com/tcmsp.php, accessed on 5 September 2024), were used to retrieve the component-related targets as well. All the targets were converted into uniform gene names using the UniProt protein database (http://www.uniprot.org/, accessed on 6 September 2024).

The target intersections were analyzed by matching the SARS-CoV-2-related targets and the AEMH-related targets. A Venn diagram was drawn using the package VennDiagram (https://cran.r-project.org/web/packages/VennDiagram/index.html, accessed on 7 September 2024). A network of medicine–components–targets–disease was drawn using the Cytoscape (version 3.9.1) software. Genes in the intersection were imported to David online database (https://david.ncifcrf.gov/tools.jsp, accessed on 8 September 2024), and the Kyoto Encyclopedia of Genes and Genomes (KEGG) enrichment and Gene Ontology (GO) analysis was conducted.

#### 4.6.2. Screening of Core Targets

For screening the core targets, the PPI network was constructed based on the genes in the intersection using the String database (http://string.embl.de/, accessed on 10 September 2024) and visualized by the Cytoscape (version 3.9.1) software. Then, the differential expressed genes between the healthy donor and patients infected with the omicron or ancestral strain were analyzed based on the transcriptome data of platelets published by Hong Wang et al. [27]. Finally, the genes obtained from PPI were intersected with the differential expressed genes, and the genes in the intersection were defined as core targets and used for verification.

### 4.7. RNA Extraction and Quantitative Real-Time PCR (qRT-PCR)

Genomic RNA was extracted from 200 µL of culture fluid and eluted into 30 µL RNase-free water using the RNeasy Mini Kit (Qiagen, Hilden, Germany) according to the manufacturer’s instructions. In the antiviral experiments, the extracted vRNA was quantitatively detected by the Nucleic Acid Detection Kit for 2019-nCoV (EDIAGNOSIS, Wuhan, China). The pseudovirus standard (High value H1) used to quantify the viral loads of the N gene of SARS-CoV-2 was purchased from FANTASIABIO. Co., Ltd. (Jinhua, China) The initial concentration of the standard is 10^7^ copies/mL. qRT-PCR was also used to validate the changes in the transcripts of the core targets selected. The extracted RNA was detected by One Step TB Green™ PrimeScript PLUS RT-PCR Kit (Perfect Real-time) (TaKaRa, Kyoto, Japan). The relative RNA expression was calculated using a classical 2^−ΔΔCt^ method after normalizing against the expression level of β-actin.

### 4.8. Molecular Docking

The 3D structures of two core target proteins, PNP (PDB ID: 1V3Q) and TPI1 (PDB ID: 6UP5), were downloaded from the RSCB PDB (https://www.rcsb.org/, accessed on 15 September 2024). The proteins were preprocessed using the PyMOL 2.3 software, including water removal and active site definition. The processed proteins were then saved in the PDB format. After that, the 26 components of AEMH were imported into Chemdraw3D (version 20.0) for energy minimization and saved as PDB files. The proteins and the components were subsequently added to hydrogens and converted to the PDBQT format with AutoDock Tools 1.5.7. Molecular docking simulations were performed with AutoDock vina (version 1.2.2). The binding mode, affinity, and key interactions were analyzed using the PyMOL 2.5.4 software.

### 4.9. Statistic Analysis

The results of the viral loads and mRNA expression levels are shown as the mean ± standard deviation. Ordinary one-way ANOVA (multiple comparisons) was used to compare the significance of differences between the means of the VC and each medicine-treated group. All statistical tests were considered statistically significant at *p* < 0.05.

## 5. Conclusions

In summary, the aqueous abstract of the herbal medicine MH has in vitro antiviral effects on SARS-CoV-2. It may exert antiviral effects by regulating the PNP and TPI1 genes in cells. Caffeic acid, rosmarinic acid, and luteolin were identified as single components with anti-SARS-CoV-2 activities from AEMH. Our results could provide detailed data and a reference for the clinical application of MH and also valuable insights for developing antiviral drugs with herbal medicines.

## Figures and Tables

**Table 1:** Components identified for AEMH by HPLC-ESI-Q-TOF/MS.

No.	Retention Time (min)	*m*/*z*	ESI	Molecular Formula	ms/ms	Compound Name
1	3.486	191.0199	[M-H]-	C6H8O7	129	Citric acid
2	4.459	188.0563	[M-H]-	C7H11NO5	128; 146	N-Acetyl-DL-glutamic acid
3	5.71	117.0191	[M-H]-	C4H6O4	-	Succinic acid
4	7.332	344.0389	[M-H2O-H]-	C10H14N5O8P	150; 211	5′-Guanylic acid
5	7.749	144.0659	[M-H]-	C6H11NO3	126	4-Acetamidobutyric acid
6	14.421	329.0517	[M-H]-	C13H14O10	153	3,4-Dihydroxybenzoic acid 3-O-β-D-Glucuronide
7	17.063	153.0194	[M-H]-	C7H6O4	109	3,4-Dihydroxybenzoic acid
8	17.897	295.0464	[M-H]-	C13H12O8	163	Caffeoyimalic acid
9	25.403	137.0237	[M-H]-	C7H6O3	-	Salicylic acid/4-Hydroxybenzoic acid
10	26.562	549.2166	[M+Hac-H]-	C22H34O12	327	Cymorcin diglucoside
11	28.462	137.0237	[M-H]-	C7H6O3	-	4-Hydroxybenzoic acid/Salicylic acid
12	40	179.0347	[M-H]-	C9H8O4	135	Caffeic acid
13	40.463	387.1647	[M+Hac-H]-	C16H24O7	165	Cymorcin monoglucoside
14	43.661	197.0447	[M-H]-	C9H10O5	179; 135	Syringic Acid
15	45.838	395.0931	[M+H-H2O]-	C17H20N2O8S	219	Zileuton O-glucuronide
16	47.368	517.2257	[M-H]-	C24H38O12	385; 161; 205	Cinnamoside
17	48.619	387.1649	[M-H]-	C18H28O9	-	-
18	50.148	239.092	[M-H]-	C12H16O5	151; 177; 195	3-Carboxy-4-methyl-5-propyl-2-furanpropionic acid
19	51.306	225.1129	[M-H]-	C12H18O4	147	Tuberonic acid
20	53.809	593.1510	[M-H]-	C27H30O15	353; 503	Luteolin 7-O-rutinoside
21	62.659	461.0708	[M-H]-	C21H18O12	285	Luteolin-3′-D-glucuronide/Luteolin-7-O-glucuronide
22	63.54	461.0706	[M-H]-	C21H18O12	285	Luteolin-3′-D-glucuronide/Luteolin-7-O-glucuronide
23	64.744	447.0914	[M-H]-	C21H20O11	285	Luteolin-7-O-glucoside
24	68.451	359.0756	[M-H]-	C18H16O8	197	Rosmarinic acid
25	69.1	445.0755	[M-H]-	C21H18O11	175; 269	Apigenin-7-O-glucuronide
26	86.106	285.0393	[M-H]-	C15H10O6	267	Luteolin
27	93.427	285.0748	[M+H]+	C16H12O5	270	5,6-Dihydroxy-7-methoxyflavone

## Data Availability

No new data were created.
